# Mechanical unloading by miniature axial flow pumps in late cardiac allograft failure due to acute rejection

**DOI:** 10.1007/s10047-021-01266-4

**Published:** 2021-04-12

**Authors:** I. A. Just, E. Potapov, C. Knosalla, F. Schoenrath

**Affiliations:** 1 Department of Cardiothoracic and Vascular Surgery, German Heart Center Berlin, Augustenburger Platz 1, 13353 Berlin, Germany; 2 DZHK (German Center for Cardiovascular Research), Partner Site Berlin, Berlin, Germany; 3 Charité-Universitätsmedizin Berlin, Corporate Member of Freie-Universität, Humboldt-Universität and Berlin-Institute-of-Health, Berlin, Germany

**Keywords:** Heart transplantation, Acute rejection, Mechanical circulatory support, Mechanical unloading

## Abstract

Allograft failure secondary to rejection commonly requires a multimodal treatment, ultimately including mechanical circulatory support. A few case reports have demonstrated the use of Impella-devices due to its assumed favorable safety profile in this fragile cohort. However, this treatment option does not play a role in choice of anti-rejective therapy in clinical routine up to date. We summarize our institutional experiences and literature mini-review on Impella-based treatment strategies in allograft rejection after heart transplantation. In all seven cases, three from our institution and four reported in the literature, Impella-based therapies led to hemodynamic stabilization in allograft failure secondary to rejection. Adverse events included hemolysis, non-fatal bleeding and in one patient a relevant aortic valve insufficiency occurred. All patients showed an improvement of allograft function. Two patients died in context of severe immunosuppression or late secondary organ failure. Based on the limited available data, we propose that Impella-mediated mechanical unloading represents a valuable option for hemodynamic stabilization in severe allograft failure due to rejection, enabling an initiation of causal therapy and thereby potentially representing an opportunity to prevent mortality. Furthermore, we hypothesize it might add to the traditional therapeutic approaches by facilitating recovery by decompressing the myocardium in allograft rejection.

## Introduction

Allograft failure (AF) due to rejection is one of the leading causes of mortality after orthotopic heart transplantation (OHT) [1]. Temporary left ventricular (LV) mechanical unloading by transcutaneous implantation of miniature axial flow pumps solely (Impella) or combined with veno-arterial extracorporeal membrane oxygenation (v-a ECMO; ECMELLA) has been successfully performed in potentially recoverable heart failure, e.g., due to acute myocarditis or infarction. The concept of prolonged Impella mediated decompression for several weeks (PROPELLA) is based on a hemodynamically beneficial reduction of afterload and facilitates disease modifying processes by reducing myocardial wall stress and thereby decreasing LV work and oxygen demand which is relevant for recovery [2]. Nevertheless, clinical experience in decompression of the transplanted heart in a post OHT cohort remains scarce.

## Patients and methods

We report three cases of late AF due to acute rejection (AR) in which we applied mechanical unloading in a bridge-to-recovery strategy and present a mini-review of the literature. The data were analyzed retrospectively according to the approval by the local ethics committee.

### Clinical cases

All patients presented with cardiogenic shock in the context of late AF due to AR differing in acuity of onset. Patient 1 was previously treated with immunosuppressant therapy including tocilizumab, which has been described once by January et al. [3], and was admitted due to cardiac decompensation (Table 1). Low cardiac output with subsequent refractory secondary organ failure led to an insertion of an Impella device. Patient 2 presented with dyspnea. Shortly after admission, need for a brief cardiopulmonary resuscitation occurred and a v-a ECMO was employed. Patient 3 was continuously cardiopulmonary resuscitated due to complete loss of LV function. A v-a ECMO was initiated. In both latter patients, the ECMO was consecutively exchanged for an Impella device.

**Table 1:** Demographics, mechanical support and outcome

Patient		1	2	3
Age (years)		28	55	31
Gender		Male	Male	Male
Transplanted (years)		1.4	1.5	6.4
Setting		Cardiogenic shock	Cardiogenic shock, CPR	Cardiogenic shock, CPR
Rejection ACR/AMR DSA Class1/2		0/0 Negative/positive	1/1 (i +) Negative/negative	0/0 Negative/negative
Causal treatment		PST, 8 × plasmapheresis, 3xIVIG (1 g/kg), 3 × ATG (1 mg/kg), 1 × rituximab (850 mg), 1 × tocilizumab (800 mg)	PST, 6 × plasmapheresis, 2xIVIG (1 g/kg), 2 × ATG (1 mg/kg), 1 × rituximab (1000 mg)	PST, 7 × plasmapheresis, 1 × ATG (1 mg/kg)
Support		PROPELLA	ECMO, PROPELLA	ECMO, Impella
Support (days)		21	8 + 44	12 + 1
Complications		Dislocation	None	None
Baseline without MCS	pH lactate ScvO_2_ CO;CI Inotropes LV-EDD/ESD LV-EF/FS	7.34 21 mmol/l 57.9% 5.3 l/min; 2 l/min/m^2*^ Milrinone 0.74 µg/kg/min Dobutamine 14.81 µg/kg/min 48/46 mm 15/4%	7.36	6.78 147 mmol/l 0 l/min;0 l/min/m^2†^ Adrenaline 0.31 µg/kg/min 5/55 mm 0/0%
			109 mmol/l	
			Adrenaline 0.06 µg/kg/min	
			58/52 mm	
			10/10%	
On Impella support (best value)	support-day^§^ Maximum-pump-flow pump-flow^§^ pH Lactate ScvO_2_ CO; CI Inotropes LV-EDD/ESD LV-EF/FS	6 4.4 l/min 3.2 l/min 7.41 13 mmol/l 72.4% 8.8 l/min; 4.5 l/min/m^2‡^ Milrinone 0.53 µg/kg/min Dobutamine 5.3 µg/kg/min 52/46 mm	32 4.9 l/min 1 l/min 7.42 6 mmol/l 60.7% 6.5 l/min; 4.1 l/min/m^2‡^ None 57/44 mm 40/23%	1 5.6 l/min 5.6 l/min 7.4 13 mmol/l 71.2% 2.9 l/min; 1.4 l/min/m^2†^ None 46/35 mm 30/24%
		30/12%		
After Impella explantation	pH lactate ScvO_2_ CO;CI inotropes LV-EDD/ESD LV-EF/FS		7.38 6 mmol/l 64.8% 4.2 l/min;2.7 l/min/m^2‡^ None 56/47 mm 30/16%	
Outcome		Sepsis	Discharge home	MOF

*ACR* acute cellular rejection (ISHLTgrade), *AMR* antibody-mediated rejection, *ATG* antithymocyte-globulin, *CO;CI* cardiac-output;cardiac-index, *CPR* cardiopulmonary resuscitation, *DSA* donor specific antibody, *ECMO* extracorporeal membrane oxygenation, *EDD/ESD* end-diasolic/end-systolic diameter, *EF* ejection fraction, *FS* fractional shortening, *IVIG* intravenous immunoglobulin, *LV* left ventricle, *MCS* mechanical circulatory support, *MOF* multiple organ failure, *PROPELLA* prolonged Impella-therapy, *PST* pulse steroid treatment, *ScvO*_*2*_ central venous oxygen saturation

*Echocardiographic (velocity–time-integral)

^†^Echocardiographic (multi-planar volumetric)

^‡^Thermodilution

^§^At time point of measurement

Impella5.5^®^ pumps (AbioMed, Danvers, USA) were inserted via 10 mm Gore-Tex-vascular-grafts anastomosed to the axillary artery. The longest duration of Impella support provided was 44 days. In this patient, myocardial recovery enabled a MitraClip procedure, which was performed under Impella support.

Our weaning considerations were based on echocardiographic investigations with a stepwise, temporary reduction of the support by decreasing the pump flow. Left and right ventricular ejection fractions, dimensions, heart valve regurgitations and hemodynamics were carefully assessed.

Ultimately, LV functions partially recovered from 0–15 to 30–40% in all patients and we observed no severe complications. In one patient dislocation of the Impella into the aortic-root and hemolysis occurred, prompting device exchange. Eventually, two patients died after partial myocardial recovery. Patient 2 was discharge home after 44 days of PROPELLA-therapy; the left ventricular ejection fraction was 41% in a routine follow-up visit 9 months after discharge.

## Discussion and mini-review of literature

In the described life-threatening constellations of all three patients, rapid installation of cardiopulmonary support was crucial for hemodynamic stabilization and enabled an initiation of anti-rejective therapies with a consecutive partial recovery of LV function.

We observed no severe complications, which was in line with the current body of the literature: a most recent clinical trial comparing ECMO and Impella in non-transplanted patients showed significantly less complications and better mid-term outcomes in the Impella-cohort. Additionally, these patients required less transfusion, a noteworthy aspect for transplanted patients reducing the risk of an induction or an increase in humoral sensitization [4].

The use of Impella2.5^®^ or Impella5.0^®^ pumps have been described in four single case reports in adults after OHT with early or late AF secondary to humoral or cellular rejection in a bridge-to-recovery strategy [5–8]. The pumps were implanted solely or in a combined approach with an intraaortic balloon pump or a TandemHeart^®^ for RV support for 2–14 days. The hemodynamic stabilization allowed an initiation of intense antirejection medication including methylprednisolone combined with antithymocyte globulin (in case of cellular rejections) or plasmapheresis, intravenous immunoglobulin and rituximab (in case of humoral rejection) (Table 2). All patients were admitted in cardiogenic shock with a rather subacute onset. In all reported cases, an at least partial recovery of the left ventricular function was observed. The described complications included one mechanical device failure after hemodynamic stabilization just before the planned Impella removal without relevant clinical consequences [6]; one induced significant aortic valve insufficiency due to a flail right coronary cusp, which required a transcatheter valve replacement [8]; and the need for multiple transfusions of blood products due to mechanical hemolysis [5] or cannula-site bleeding [7]. Latter occurred based on a thrombocytopenia, which was not clearly referred to be mechanically induced by either the Impella or TandemHeart device. Moreover, a concomitant coagulopathy was reported. Its management required device removal after the adjustment of the heparin dosing (target activated clotting time of < 150 s) and stopping heparin for 24 h did not lead to hemostasis. No thrombotic events occurred. Furthermore, despite aggressive immunosuppression, no device-associated infection was reported. All patients were discharged.

**Table 2:** Cases of Impella use in adults with allograft failure after transplantation

		Samoukovic et al. [5]	Beyer et al. [6]	Rajagopal et al. [7]	Chandola et al. [8]
Age (years); gender		52; female	64; male	36; male	45; female
Transplanted (years)		5.5	5	2	0.3
Setting		cardiogenic shock	cardiogenic shock, CPR	cardiogenic shock	cardiogenic shock
Rejection ACR/AMR		2R/−	1R/ +	Presumably/−	Yes/−
Antirejection medication		ATG, MP, MMF	MP, PP, IVIG, rituximab	ATG, MP	No data published
Support		IABP + Impella5.0	Impella2.5	Impella2.5 + TandemHeart	Impella5.0
Insertion		Femoral			femoral
Support (days)		2 + 7	2	9 + 5	14
Concept		Bridge-to-recovery	Bridge-to-recovery	Bridge-to-recovery	Bridge-to-recovery
Complications		hemolysis	device failure	cannula-site bleeding	Severe aortic insufficiency
Baseline without Impella	NTproBNP pH; lactate CI;PCWP SmvO_2_ isotropes LV-EF	7.23; 12.3 mmol/l < 1.2 l/min/m^2^ dobutamine, adrenaline 10%	5814 pg/ml 1.8 l/min/m^2^; 26 mmHg dopamine, dobutamine 39%	1080 ng/l 1.2 l/min/m^2^;28 mmHg 34% milrinone 10%	1.6 l/min/m^2^;28 mmHg Dobutamine, dopamine, epinephrine 23%
On Impella support	Maximum pump-flow CI;PCWP SmvO_2_ LV-EF	5.0 l/min 40%	2.0 l/min 2.5 l/min/m^2^	2.4 l/min 2.6 l/min/m^2^; 12 mmHg 67%	l/min/m^2^; 16 45%
After Impella removal	LV-EF	50%	41%	40% (55%^a^)	No data published
Overall outcome		Discharge	Discharge	Discharge	Asymptomatic*

*ACR* acute cellular rejection (ISHLTgrade), *AMR* antibody-mediated rejection, *ATG* antithymocyte globulin, *CI* cardiac-index, *CPR* cardiopulmonary resuscitation, *EF* ejection fraction, *IVIG* intravenous immunoglobulin, *LV* left ventricle, *MMF* mycophenolate mofetil, *MP* methylprednisolone, *PCWP* pulmonary capillary wedge pressure, *PP* plasmapheresis, *SmvO*_*2*_ mixed venous oxygen saturation

*At 6–9 months follow-up

In our cohort, two of three patients died in context of an infection while on intense immunosuppression and due to late secondary organ failure following prolonged ischemia. In contrast, all patients reported by single case reports survived [5–8]. A retrospective comparison of the non-standardized settings is speculative, especially since data on hemodynamics are not stated for all patients. Possibly, on one hand, the AR in our cohort was more severe; all three patients showed an advanced secondary organ failure with limited chances of survival on admission. On the other hand, the reported single cases might not have been published if the patients would have died.

Furthermore, biventricular Impella pump employments (Impella CP and RP, BiPella) were recently described in two case series of pediatric transplanted patients with a comparable profile of adverse events, including bleeding, hemolysis and femoral arterial dissection [9, 10].

## Conclusion

Our experiences and the mini-review of literature lead us to assume that an Impella-mediated circulatory support represents a valuable treatment option for hemodynamic stabilization, enabling an initiation of causal therapy, which leads to an allograft recovery and possibly prevents mortality. In the future, a preventive approach with lowering the threshold for the decision of initiating LV decompression and opening up for a shift in concept of early mechanical circulatory support, for example, before onset of secondary organ failure, is potentially conceivable in allograft rejection. However, large-scale clinical research and evidence is needed to confirm these hypotheses.

## References

[1] Chambers DC, Cherikh WS, Harhay MO (2019). The International Thoracic-Organ-Transplant-Registry of the International Society for Heart and Lung Transplantation: thirty-sixth adult heart transplantation report-2019. JHLT.

[2] Tschoepe C, VanLinthout S, Klein O (2019). Mechanical unloading by fulminant myocarditis: LV-IMPELLA, ECMELLA BI-PELLA and PROPELLA concepts. J CardiovascTransl Res.

[3] January S, Pottebaum A, Raymer D (2019). Tocilizumab for antibody-mediated rejection in the setting of cardiac allograft vasculopathy. JHLT.

[4] Karami M, den Uil CA, Ouweneel DM (2020). Mechanical circulatory support in cardiogenic shock from acute myocardial infarction: Impella CP/5.0 versus ECMO. Eur Heart J Acute Cardiovasc Care.

[5] Samoukovic G, Al-Atassi T, Rosu C (2009). Successful treatment of heart failure due to acute transplant rejection with the Impella LP 5.0. Ann ThoracSurg.

[6] Beyer AT, Hui PY, Haeusslein E (2010). TheImpella 2.5 L for percutaneous mechanical circulatory support in severe humoral allograft rejection. J Invasive Cardiol.

[7] Rajagopal V, Steahr G, Wilmer CI (2010). A novel percutaneous mechanical biventricular bridge to recovery in severe cardiac allograft rejection. JHLT.

[8] Chandola R, Cusimano R, Osten M (2012). Severe aortic insufficiency secondary to 5L Impella device placement. J Card Surg.

[9] Lasa JJ, Castellanos DA, Denfield SW (2018). Acute biventricular percutaneous Impella ventricular assist device use in pediatric patients. ASAIO J.

[10] Ankola AA, McAllister J, Turner ME (2020). Biventricular Impella use in pediatric patients with severe graft dysfunction from acute rejection after heart transplantation. Artif Org.

